# A straightforward approach to designing a scoring system for predicting length-of-stay of cardiac surgery patients

**DOI:** 10.1186/1472-6947-14-89

**Published:** 2014-10-13

**Authors:** Paolo Barbini, Emanuela Barbini, Simone Furini, Gabriele Cevenini

**Affiliations:** Department of Medical Biotechnologies, University of Siena, Viale Bracci 53100, Siena, Italy; Health-Care Management, University Hospital of Siena, Siena, Italy

**Keywords:** Intensive care unit, Decision-support system, Prediction model, Scoring system

## Abstract

**Background:**

Length-of-stay prediction for cardiac surgery patients is a key point for medical management issues, such as optimization of resources in intensive care units and operating room scheduling. Scoring systems are a very attractive family of predictive models, but their retraining and updating are generally critical. The present approach to designing a scoring system for predicting length of stay in intensive care aims to overcome these difficulties, so that a model designed in a given scenario can easily be adjusted over time or for internal purposes.

**Methods:**

A naïve Bayes approach was used to develop a simple scoring system. A set of 36 preoperative, intraoperative and postoperative variables collected in a sample of 3256 consecutive adult patients undergoing heart surgery were considered as likely risk predictors. The number of variables was reduced by selecting an optimal subset of features. Scoring system performance was assessed by cross-validation.

**Results:**

After the selection process, seven variables were entered in the prediction model, which showed excellent discrimination, good generalization power and suitable sensitivity and specificity. No significant difference was found between AUC of the training and testing sets. The 95% confidence interval for AUC estimated by the BCa bootstrap method was [0.841, 0.883] and [0.837, 0.880] in the training and testing sets, respectively. Chronic dialysis, low postoperative cardiac output and acute myocardial infarction proved to be the major risk factors.

**Conclusions:**

The proposed approach produced a simple and trustworthy scoring system, which is easy to update regularly and to customize for other centers. This is a crucial point when scoring systems are used as predictive models in clinical practice.

## Background

Prediction models are increasingly important in clinical practice, as indicated by the number of recent publications describing their development. One of their purposes is to aid clinical decision-making by combining patient characteristics in order to estimate the probability of a certain disorder or problem (diagnosis and prognosis). In particular, prognostic models are widely accepted in intensive care units (ICUs) for predicting outcome of critical patients [1–6]. In many cases, these models are scoring systems in which the predictor variables are usually selected and scored subjectively by expert consensus or objectively using statistical methods [7, 8].

While mortality can be considered the primary outcome, over the years technological advances have led to a significant decrease in mortality for certain patient populations, for example cardiac surgery patients. In these cases, morbidity or prolonged stay in intensive care have been suggested as valid end points and more attractive targets for developing operative risk models. In particular, models that estimate the length-of-stay (LOS) in ICU of cardiac surgery patients can be very useful for internal purposes. Reliable prediction of LOS is the starting point for good internal management of operating rooms.

When a prediction system is developed primarily for internal purposes, such as operating-room scheduling, the model should not only be simple, reliable and characterized by high sensitivity/specificity, but also easy to modify, so that clinicians can customize it to their specific patient subpopulation and update it with new data sets. Unfortunately, retraining and updating are critical points of scoring systems, because the design and development of scoring systems generally imply theoretical modeling ability and statistical procedures seldom available in a clinical environment, and make it complicated to modify a given model. Thus, in clinical practice, scoring systems are usually used in their original standard form, as developed with training data from different countries and/or centers. If data from the specific scenario is not considered during model training, there can be significant loss in model performance [9].

Model customization is essential when it is difficult to standardize local practices and patient populations differ [9–13]. Easy updating is another crucial feature. In fact, acquisition of new, correctly classified patients enables the training set to be increased day by day, improving model performance in a corresponding way. Progress in medical techniques also makes it necessary to be able to update the model continuously. It is therefore fundamental to use approaches allowing the decision rule to be derived in a straightforward manner so that it is easily modified, locally customized, updated and validated.

In the present study, a scoring system was designed to predict prolonged stay in intensive care after heart surgery, using a straightforward approach recently proposed [14]. It is based on the naïve Bayes rule [15], which generally shows good classification accuracy, even when the assumption of independence does not hold [16–18]. Although the prediction model was trained using a sample of patients who underwent heart surgery in a specific institution, it can be modified directly to customize it for other centers.

## Methods

### Scoring-system development and validation

The scoring system was developed using a simple approach recently proposed [14], which uses the well-known Bayes rule assuming that features are all conditionally independent of each other given the class. This strong (naïve) assumption drastically simplifies the problem of estimation from training data.

Given an N-dimensional observation vector **x** = (*x*_1_, *x*_2_, ….., *x*_N_) and two patient classes *ω*_1_ and *ω*_2_ (adverse and positive outcome, respectively), the decision rule was written as
1

where *P*(*ω*_*i*_) is the *a priori* probability of class *ω*_*i*_ (i = 1,2) and *w*_*xj*_ (*j* = 1,2,….,N) are log-likelihood ratios, which can be calculated directly from data acquired in any specific institution.

We chose this type of scoring system because it is easily customized to any specific scenario and it also can be easily updated by entering new and removing older data (for more details see Ref. [14]).

After selecting the subset of features to include in the predictive model, scoring system performance was assessed by five-fold cross-validation, randomly dividing the sample into five roughly equal non-overlapping subsamples. The whole validation process required five rounds, with each of the five subsamples used exactly once as testing data. In particular, in each round, a single subsample was retained as the validation data for testing the model, and the remaining four subsamples were used as training data to estimate the weight of each feature in the scoring system. This allowed us to assess the performance of the scoring system when its parameters (weights) were estimated on datasets different from testing data.

AUC and its 95% confidence interval were calculated in the training and testing sets. In particular, the bias-corrected and accelerated (BCa) bootstrap method was used to estimate the 95% confidence intervals of AUC, using one thousand bootstrapped samples generated from original data [19].

The prior probabilities *P*(*ω*_1_) and *P*(*ω*_2_) were both assumed to be 0.5, so that the threshold value in equation 1 was set at zero. All computations were done using IBM SPSS Statistics (IBM Corp., Armonk, New Yok, USA) and MATLAB (The MathWorks, Inc., Natick, Massachusetts, USA) code.

### Study population and feature selection

The data set for developing the locally customized scoring system was retrieved from the computerized database of the Department of Medical Biotechnologies of Siena University. Due to the retrospective nature of the study, the need for informed consent was waived. The authors did not have direct access to this institutional database. Aggregate patient data was provided anonymously. Use of anonymous aggregate data is consented because it does not implicate the privacy concerns that apply to patient-identifiable information. The study was undertaken after approval of the Ethics Committee (Comitato etico locale e comitato etico per la sperimentazione clinica dei medicinali) of Siena University Hospital and was conducted in compliance with the Helsinki declaration.

A sample of consecutive adult patients who underwent heart surgery between 2000 and 2007 was used. Exclusion criteria included operation without cardiopulmonary bypass, heart or heart-lung transplant, aortic dissection, age less than 18 years and death. Some records (about 1.5%) were excluded from the analysis because they contained insufficient data to design the classifier. The final size of the sample used in the study was 3256 patients. These patients underwent isolated coronary artery bypass grafting (CABG) and isolated valve or combined procedures (CABG plus valve) at the Cardiac Surgery Unit of Siena University Hospital, Italy.

Length of stay in the ICU was chosen as outcome. Adverse outcome was defined as LOS greater than or equal to 5 days (i.e. 120 hours), and normal LOS was defined as less than 5 days. The mean and standard deviation of LOS were 68 and 112 hours, respectively.

A collection of 36 preoperative, intraoperative and postoperative dichotomous variables and 16 non dichotomous (continuous or discrete) variables was considered *a priori* as a wide set of features for predicting patient outcome on the basis of clinical judgment and past experience [13]. Preoperative and intraoperative data was collected under the anaesthesiologist’s supervision. Postoperative data was collected in the first three hours after admission to the ICU.

To lower the bias of the naïve independence assumption, the above number of variables was reduced by a procedure aimed at selecting an optimal subset of features to include in the predictive model. Firstly, the discrimination power of each variable was evaluated individually to eliminate less important features once and for all. For this purpose we calculated the 99% confidence intervals of the odds ratio [20] for each dichotomous variable and for other variables, dichotomized on the basis of their medians (cut-off point). Only variables with an odds ratio significantly different from 1 (p < 0.01) were chosen as potential competing features to be taken into consideration for the final stepwise selection, using the receiver operating characteristic (ROC) curve [21]. The direction of search proceeded in a forward manner [22] and, at each step of the algorithm, the variable giving the best increase in area under the ROC curve (AUC) was entered in the model [23]. To decrease the chance of entering redundant features that might introduce dependencies, the criterion for halting the search process was slightly less conservative than the one suggested in previous papers [22, 23]: the procedure was stopped when the cumulative increment in AUC obtained in three consecutive steps was less than 1%.

## Results

Table 1 shows the 99% confidence interval of the odds ratio for the whole set of 36 preoperative, intraoperative and postoperative dichotomous variables chosen *a priori* on the basis of clinical judgment and past experience. Table 2 shows the cut-off (median on original sample) and 99% confidence interval of the odds ratio for the 16 non dichotomous variables. Seventeen dichotomous and five non dichotomous variables (in italics in Tables 1 and 2) were eliminated from the subsequent stepwise selection since their corresponding 99% confidence interval of the odds ratio included 1. The remaining variables, whose odds ratios were significantly different from 1 (p < 0.01), were considered in the stepwise selection process (nineteen dichotomous and eleven continuous variables, which were discretized into 4 categories, according to their value falling into the 1st, 2nd, 3rd or 4th quartile interval).

**Table 1:** **The whole set of dichotomous preoperative, intraoperative and postoperative variables considered** ***a priori*** **as likely predictors**

Variable	99% confidence interval of odds ratio
*Gender*	0.659-1.21
Emergency	1.44-6.57
*Urgency*	0.970-2.39
*Blood hypertension*	0.987-1.87
*Treated diabetes*	0.884-1.70
Acute myocardial infarction	1.57-6.38
*Recent myocardial infarction (<7 days)*	0.663-2.96
Preoperative intra-aortic balloon pump	2.79-9.63
*Tachycardia*	0.885-5.47
Cardiogenic shock	3.15-21.85
*Cardiac massage*	0.988-21.62
*Endocarditis*	0.394-8.77
*Unstable angina*	0.729-1.47
Heart failure	1.90-5.25
Chronic dialysis	3.51-36.70
*Previous cerebrovascular events*	0.998-3.01
Chronic obstructive pulmonary disease	1.07-2.80
Anti-platelet drugs	0.545-0.984
Dicumarole therapy	1.39-4.18
*Heparin therapy*	0.873-1.57
*Thrombolytic therapy*	0.125-3.88
Combined cardiovascular surgery	2.19-4.21
Repeated cardiac surgery	1.63-4.68
*Aortocoronary bypass*	0.712-1.32
Plastic valve prosthesis	1.48-3.14
Valve substitution	1.22-2.72
*Plastic surgery for aortic valve*	0.241-8.86
Aortic valve substitution	1.06-1.96
Plastic surgery for tricuspid valve	1.50-7.70
Surgical procedure different from isolated coronary artery bypass graft	1.52-2.74
*Aortic arch pathology*	0.776-4.30
*Carotid endarterectomy*	0.382-2.55
Addition of blood to cardiopulmonary bypass circuit	1.96-3.59
Use of fresh frozen plasma	2.24-4.11
*Significant bleeding (>100 ml)*	0.728-1.39
Low postoperative cardiac output	12.3-23.9

The variables in italics were eliminated from the model because their corresponding odds ratios were not significantly different from 1 (p < 0.01).

**Table 2:** **The whole set of non dichotomous preoperative, intraoperative and postoperative variables considered** ***a priori*** **as likely predictors**

Variable (units)	99% confidence interval of odds ratio	Cut-off
Age (years)	1.36-2.46	69
*Height* (cm)	0.668-1.19	167
*Weight* (kg)	0.587-1.05	71
Body surface area (m^2^)	0.550-0.983	1.8
NYHA heart failure classification	1.55-2.78	2
Preoperative hematocrit (%)	0.499-0.895	39.2
Preoperative creatinine (mg/dl)	1.51-2.69	1.0
*Preoperative bilirubin* (mg/dl)	0.812-1.45	0.8
ECC time (min)	1.75-3.22	122
Aortic clamping time (min)	1.44-2.61	86
Minimum hematocrit at cardiopulmonary bypass (%)	0.517-0.924	24
Minimum intraoperative temperature (°C)	0.410-0.743	32.5
*Postoperative hematocrit* (%)	0.570-1.02	31
*Postoperative venous O*_*2*_ *saturation* (%)	0.656-1.17	63.7
Postoperative creatinine (mg/dl)	3.35-6.29	1.2
Postoperative bilirubin (mg/dl)	1.99-3.70	1

NYHA = New York Heart Association; ECC = extracorporeal circulation.

The 99% confidence intervals of the odds ratio were calculated after dichotomizing each variable on the basis of the cut-off (median calculated on original sample). The variables in italics were excluded from the model because their corresponding odds ratios were not significantly different from 1 (p < 0.01).

The stepwise process selected seven variables, three of which were dichotomous (low postoperative cardiac output, preoperative chronic dialysis and acute myocardial infarction). The detailed results obtained step-by-step are summarized Table 3.

**Table 3:** **Results obtained by the stepwise process of variable selection**

Step number	Variable entered	AUC value
1	Low postoperative cardiac output	0.7442
2	Postoperative creatinine	0.8342
3	Extracorporeal circulation time	0.8507
4	Age	0.8551
5	Postoperative bilirubin	0.8589
6	Chronic dialysis	0.8612
7	Acute myocardial infarction	0.8631

The 95% confidence interval for AUC estimated by the BCa bootstrap method in the training test was [0.841, 0.883] and the median AUC was 0.863. No significant difference was found when estimating AUC in the testing set, where the median and 95% confidence interval were 0.859 and [0.837, 0.880], respectively. On the basis of the Hosmer-Lemeshow rule [24] an AUC greater than 0.8 indicated excellent discrimination, while the absence of significant differences between the results obtained with the training and testing sets denoted good generalization power.

Table 4 shows the confusion matrix obtained with the testing set. The first row of the matrix refers to patients with LOS less than 5 days (normal outcome), and the second to patients with LOS greater than or equal to 5 days (adverse outcome). 2403 patients with normal outcome and 268 patients with adverse outcome were correctly classified, giving an overall correct-classification of 82%. Of course, the values in the table can be interpreted as true negatives, false positives, false negatives and true positives, so that the correct classification percentage of patients with normal outcome corresponds to the specificity (SP), while the correct classification percentage of patients with adverse outcome represents the sensitivity (SE) of the model, i.e. SP = 83%, SE = 74%.

**Table 4:** **Confusion matrix and correct-classification percentages obtained with the testing set**

		Predicted class		Correct classification percentage
		LOS < 5 days	LOS ≥ 5 days	
Actual class	LOS < 5 days	2403	492	83% (SP)
	LOS ≥ 5 days	93	268	74% (SE)
Overall correct-classification percentage: 82%				

SP = Specificity, SE = Sensitivity.

Tables 5 and 6 show the weights of the selected dichotomous and non dichotomous features in the scoring system, respectively. Since the highest positive weight in the study sample was assigned to chronic dialysis, patients in chronic dialysis showed a considerable risk of prolonged length of stay in the intensive care unit after heart surgery in the scenario considered. Important risk factors were also a low postoperative cardiac output and acute myocardial infarction. Table 6 also shows that low blood concentrations of postoperative creatinine and bilirubin were significant protective factors.

**Table 5:** **Weights of the selected dichotomous features in the scoring system**

	No	Yes
Low postoperative cardiac output	-0.7418	2.0920
Chronic dialysis	-0.0305	2.3647
Acute myocardial infarction	-0.0692	1.5864

**Table 6:** **Weights of the selected continuous features in the scoring system**

	1 ^st^quartile interval	2 ^nd^quartile interval	3 ^rd^quartile interval	4 ^th^quartile interval
Postoperative creatinine	-0.8848	-0.7004	-0.0678	1.0344
Extracorporeal circulation time	-0.6738	-0.3335	-0.1140	0.7261
Age	-0.5917	-0.1154	-0.0278	0.5111
Postoperative bilirubin	-0.7184	-0.4112	-0.0163	0.7972

An analysis of Table 6 shows that the weights corresponding to each non dichotomous feature monotonically increase from the first to the fourth quartile interval. However, this increase is generally quite nonlinear. For example, the weights corresponding to postoperative creatinine values in the first and second quartile intervals differ little from each other (-0.88 vs. -0.70), but change drastically in the third (-0.07) and fourth quartile intervals (1.03). This result shows that postoperative creatinine values below the median can be considered a protective factor, whereas values in the fourth quartile represent a risk factor. Finally, the weight of creatinine values in the third quartile interval is close to zero.

## Discussion

Outcome prediction is a key point in ICUs, not only for prognosis assessment, but also for cost-benefit analysis, health-care management, comparisons between centers, monitoring/assessment of new therapies and population sample comparison studies. A distinction must be made between predictive models for mortality and predictive models for LOS. For the former task, stable benchmarks are needed to conclude whether high-quality care is being delivered across institutions. On the contrary, for LOS, a customized model can be useful for internal healthcare management purposes.

In many cases the predictive models are scoring systems, in which the predictor variables are usually selected and scored subjectively by expert consensus or objectively using statistical methods. These systems are generally preferred by clinicians and health operators because they are so simple that individual scores can be assessed immediately, without using any data processing system. However, a common weakness of scoring systems is that their updating or customization to new populations is often not an easy task [25]. Scoring systems are therefore generally used in their original formulation also for internal management purposes, which implies a significant loss in performance, because model performance deteriorates over time or when applied to populations different from the ones on which they were developed [26].

The approach we used in the present study to get around this critical point was to derive the scoring system directly from a naïve Bayes classifier, using discrete predictors. This approach was not only straightforward but also successful, because naïve Bayes classifiers identify the parameters required for accurate classification using less training data than many other classifiers. This makes them particularly effective for datasets containing many features. Previous papers have also demonstrated that naïve Bayes classifiers may outperform more complex classification methods and show good average performance in terms of classification accuracy, especially over data sets having features that are not strongly correlated [22]. Of course this does not mean that the naïve Bayes technique is the best approach for supervised classification problems. More sophisticated models (which do not rely on the conditional independence assumption and incorporate interaction terms) may perform better. Unfortunately, sophisticated models are rarely used in clinical practice because they may be difficult to fine-tune. For example, the actual interaction terms are not easy to imagine and their choice is often heuristic. Thus, the naïve approach seems to be a satisfactory compromise between good performance and simplicity.

Problems may arise if there are several redundant predictive features, in which case a naïve Bayes classifier may show low asymptotic accuracy. Under such conditions, Langley and Sage showed that a selective naïve Bayes classifier, using an optimal subset of selected features for making predictions, sharply improved classifier performance [22]. Unfortunately, if the number N of acquired features is high, an exhaustive search of the best subset of features may be impractical. In fact, to consider all possible subsets of *h* features (*h* = 1,2,…,N), it is necessary to analyze 2^N^–1 subsets. In the present case (52 acquired variables), this is about 4.5 × 10^15^ possible subsets of variables. To solve this problem, we used a heuristic approach consisting of two steps. First we reduced the number of variables, keeping only features giving an odds ratio significantly different from 1 (p < 0.01), after dichotomisation. This allowed us to eliminate a range of variables *a priori*, thus decreasing the number of possible subsets of likely predictors. The final selection was performed by a forward search, entering the variable giving the best increase in area under the ROC curve, step-by-step in the model, and stopping the search process when the increment in AUC became negligible. This procedure regards a methodology specifically designed to develop a selective naïve classifier [22, 23]. Although the approach used in the present paper does not ensure an exhaustive search of the best subset of independent predictors, it considers all local changes to the current set of features and makes an optimal selection.

Although alternative methods of variable selection could be used, we chose a simple approach that exploits the naïve Bayes model. In particular, we judged it inappropriate to reduce the number of predictor variables by procedures based on different models (e.g. stepwise logistic regression analysis) and then use the selected set of variables in the naïve Bayes model. The type of model may influence the optimal subset of predictor variables.

Prior probabilities were assumed identical for the two classes, i.e. *P*(*ω*_1_) = *P*(*ω*_2_) = 0.5. Such a choice is often made when it is impossible or inappropriate to make use of *a priori* knowledge, even if information from available data and/or expert beliefs could be used to make these probabilities more distinctive. Actually, each change in prior probabilities is equivalent to modifying the cost of a wrong decision. Objective criteria could be used to optimize economic and social costs related to correct and false classifications. Unfortunately, despite of the importance of this goal, this type of criterion is rarely used in problems of clinical decision-making, because the costs are generally difficult to estimate and actual prevalence cannot be easily assessed.

The results showed that the scoring system derived from the naïve Bayes classifier had excellent discrimination and good generalization power. In particular the 95% confidence interval for AUC estimated by the BCa bootstrap method was [0.841, 0.883] and [0.837, 0.880] in the training and testing sets, respectively. To assess the actual performance of the scoring system, the results were compared with those obtained by a logistic regression (LR) model and a quadratic Bayesian (QB) classifier. Both models were designed on the same data set using IBM SPSS Statistics and MATLAB code.

For the LR model, the stepwise procedure of variable selection again chose seven predictors, four of which (low postoperative cardiac output, postoperative creatinine, age and postoperative bilirubin) were identical to those chosen for the scoring system. The 95% confidence intervals for AUC estimated by the BCa bootstrap method were [0.840, 0.883] and [0.833, 0.876] in the training and testing sets, respectively. No evident difference was observed between the results obtained with the scoring system and the LR model.

The QB classifier selected four predictor variables, three of which (low postoperative cardiac output, postoperative creatinine and age) were identical to those chosen for the scoring system and LR model. The last predictor variable entered in the QB classifier was aortic clamping time. It may be interesting to note that the latter variable was not present in the scoring system, which instead included extracorporeal circulation time. The number of predictor variables of the QB classifier was smaller than that of the other two classifiers (4 vs. 7). This confirms what we pointed out in previous papers, namely that the quadratic Bayesian classifier generally requires fewer predictor variables than other models [6, 13]. For the QB classifier, the 95% confidence intervals for AUC were [0.834, 0.877] and [0.829, 0.873] in the training and testing sets, respectively. Like the LR model, the QB classifier provides performance that completely overlaps with the scoring system.

The finding that more complex classification systems (LR model and QB classifier) did not give better performance than the naïve Bayes classifier suggests that the assumption of conditional independence of the selected variables was mostly true, and small deviations from the assumption did not cause significant deterioration of model performance.

Length of stay in the ICU was chosen as endpoint for this study because it is a limiting factor for operating theatre utilization for heart surgery and consequently a major parameter of cost-effectiveness. While 120 hours is a high value of LOS for cardiac surgery patients, in the example under consideration we chose this cut-off because it identifies the group of patients (about 10%) that mostly influences internal management decisions in the scenario considered.

A recent study sought to identify and validate existing prediction models for prolonged intensive care after heart surgery [27] through systematic review of the literature. It also tested several models on a large registry database comprising 11,395 heart operations. The study proved that several models showed acceptable discrimination, but no model achieved excellent discrimination. The best performance was obtained by the Parsonnet model (AUC = 0.75), followed by the European system for cardiac operative risk evaluation (AUC = 0.71). A similar AUC value was obtained by us when we used the Cleveland scoring system [3] to predict morbidity risk after cardiac surgery in our specific scenario [12]. These AUC values are somewhat distant from those obtained by the scoring system proposed in the present study. This confirms that careful model customization is indispensable for good performance, because standardization of local practices is difficult and patient populations may differ.

## Conclusion

Scoring systems are often used in ICUs to predict outcomes of critical patients. Despite their simple application, they are generally difficult to update with new sets of data and to tune to clinical institutions different from those in which they were designed. This weakness may have a negative effect on the reliability of these attractive predictive models, since the performance of models that were originally efficient may deteriorate significantly with changes in clinical scenario.

The naïve Bayes approach used in the present paper seems to overcome this difficulty, because the scoring system is completely defined by descriptive tables that are easily calculated and/or updated using data acquired in any specific institution. Although the model described in the present paper is a working example, the results obtained indicate performance very similar to a logistic regression model and a quadratic Bayesian classifier, as well as greater ease of handling.

In conclusion, although the proposed scoring system can be regarded as an objective ICU discharge model or predictive tool in the particular scenario analysed, the results demonstrate that the present approach produces a very simple and trustworthy scoring system that is easily updated and customized for other centers. This is a key message, because simple, precise customization and updating not only ensure better model performance, but also better acceptance by surgeons and anaesthesiologists.

## References

[1] Teasdale G, Jennett B (1974). Assessment of coma and impaired consciousness. A practical scale. Lancet.

[2] Knaus WA, Wagner DP, Draper EA, Zimmerman JE, Bergner M, Bastos PG, Sirio CA, Murphy DJ, Lotring T, Damiano A (1991). The APACHE III prognostic system. Risk prediction of hospital mortality for critically ill hospitalized adults. Chest.

[3] Higgins TL, Estafanous FG, Loop FD, Beck GJ, Lee JC, Starr NJ, Knaus WA, Cosgrove DM (1997). ICU admission score for predicting morbidity and mortality risk after coronary artery bypass grafting. Ann Thorac Surg.

[4] Metnitz PGH, Moreno RP, Almeida E, Jordan B, Bauer P, Campos RA, Iapichino G, Edbrooke D, Capuzzo M, Le Gall JR, SAPS 3 Investigators (2005). SAPS 3 – From evaluation of the patient to evaluation of the intensive care unit. Part 1: Objectives, methods and cohort description. Intensive Care Med.

[5] Moreno RP, Metnitz PGH, Almeida E, Jordan B, Bauer P, Campos RA, Iapichino G, Edbrooke D, Capuzzo M, Le Gall JR, SAPS 3 Investigators (2005). SAPS 3 – From evaluation of the patient to evaluation of the intensive care unit. Part 2: Development of a prognostic model for hospital mortality at ICU admission. Intensive Care Med.

[6] Barbini E, Cevenini G, Scolletta S, Biagioli B, Giomarelli P, Barbini P (2007). A comparative analysis of predictive models of morbidity in intensive care unit after cardiac surgery – Part I: model planning. BMC Med Informat Decis Making.

[7] Higgins TL (2007). Quantifying risk and benchmarking performance in the adult intensive care unit. J Intensive Care Med.

[8] Afessa B, Keegan MT (2007). Predicting mortality in intensive care unit survivors using a subjective scoring system. Crit Care.

[9] Murphy-Filkins R, Teres D, Lemeshow S, Hosmer DW (1996). Effect of changing patient mix on the performance of an intensive care unit severity-of-illness model: how to distinguish a general from a specialty intensive care unit. Crit Care Med.

[10] Schaufer JH, Maurer A, Jochimsen F, Emde C, Wegscheider K, Arntz HR, Heitz J, Krell-Schroeder B, Distler A (1990). Outcome prediction models on admission in a medical intensive care unit: do they predict individual outcome?. Crit Care Med.

[11] Ryan TA, Rady MY, Bashour CA, Leventhal M, Lytle M, Starr NJ (1997). Predictors of outcome in cardiac surgical patients with prolonged intensive care stay. Chest.

[12] Biagioli B, Scolletta S, Cevenini G, Barbini E, Giomarelli P, Barbini P (2006). A multivariate Bayesian model for assessing morbidity after coronary artery surgery. Crit Care.

[13] Cevenini G, Barbini E, Scolletta S, Biagioli B, Giomarelli P, Barbini P (2007). A comparative analysis of predictive models of morbidity in intensive care unit after cardiac surgery – Part II: an illustrative example. BMC Med Informat Decis Making.

[14] Barbini P, Cevenini G, Furini S, Barbini E (2014). A naïve approach for deriving scoring systems to support clinical decision making. J Eval Clin Pract.

[15] Mitchell TM (1997). Machine Learning.

[16] Domingos P, Pazzani M (1997). On the optimality of the simple Bayesian classifier under zero–one loss. Mach Learn.

[17] Lavrac N (1998). Intelligent data analysis for medical diagnosis: using machine learning and temporal abstraction. AI Comm.

[18] Demichelis F, Magni P, Piergiorgi P, Rubin MA, Bellazzi R (2006). A hierarchical Naïve Bayes Model for handling sample heterogeneity in classification problems: an application to tissue microarrays. BMC Bioinformatics.

[19] DiCiccio TJ, Efron B (1996). Bootstrap confidence intervals. Stat Sci.

[20] Armitage P, Berry G, Matthews JNS (2002). Statistical Methods in Medical Research.

[21] Lasko TA, Bhagwat JG, Zou KH, Ohno-Machado L (2005). The use of receiver operating characteristic curves in biomedical informatics. J Biomed Inform.

[22] Langley P, Sage S, de Mantaras RL, Pool D (1994). Induction of Selective Bayesian Classifiers. Proceedings of the Tenth Conference on Uncertainty in Artificial Intelligence.

[23] Boullé M, Guyon I, Gunn S, Nikravesh M, Zadeh LA (2006). An Enhanced Selective Naïve Bayes Method with Optimal Discretization. Feature Extraction – Foundations and Applications.

[24] Hosmer DW, Lemeshow S (2000). Applied Logistic Regression.

[25] Kuzniewicz MW, Vasilevskis EE, Lane R, Dean ML, Trivedi NG, Rennie DJ, Clay T, Kotler PL, Dudley RA (2008). Variation in ICU risk-adjusted mortality: impact of methods of assessment and potential confounders. Chest.

[26] Kramer AA (2005). Predictive mortality models are not like fine wine. Crit Care.

[27] Ettema RGA, Peelen LM, Schuurmans MJ, Nierich AP, Kalkman CJ, Moons KGM (2010). Prediction models for prolonged intensive care unit stay after cardiac surgery: systematic review and validation study. Circulation.

[CR28] The pre-publication history for this paper can be accessed here:http://www.biomedcentral.com/1472-6947/14/89/prepub

